# Industrial Anomaly Detection and Fault Grade Assessment for Railway Catenary Components Based on Diffusion Models

**DOI:** 10.3390/s26154783

**Published:** 2026-07-28

**Authors:** Hongyue Qian, Zhiwei Han, Weijia Hong, Haonan Yang, Hui Wang, Jilin Li, Zhigang Liu

**Affiliations:** The School of Electrical Engineering, Southwest Jiaotong University, Chengdu 610031, China; qhy020526@163.com (H.Q.); hongweijia0619@163.com (W.H.); hut19960710@163.com (H.Y.); wanghui163@my.swjtu.edu.cn (H.W.); 13348846036@163.com (J.L.); liuzg_cd@126.com (Z.L.)

**Keywords:** railway catenary, industrial anomaly detection, diffusion-based modeling, fault severity assessment, multi-physics coupling

## Abstract

As a critical component of electric railways, catenary systems are prone to cracks, loosening, corrosion, and wear under long-term vibration, fatigue, and environmental erosion. However, ambiguous fault boundaries, large inter-component variations, and tiny defects severely hinder reliable anomaly detection and condition assessment. To address these challenges, this paper proposes a vision-based intelligent fault assessment framework for railway catenary components based on a novel Railway Diffusion-based Anomaly Detection (Rail-DiffAD) model. Specifically, Rail-DiffAD combines residual feature mapping, a Multi-scale Partial Convolutional Spatial-Channel Attention (MPSCA) module with Log-Barrier Bi-directional Constraint Loss (LBBCL), and conditional diffusion-based distribution modeling to achieve robust anomaly localization in complex industrial scenarios. Furthermore, a severity-aware diffusion representation is introduced to characterize structural defect evolution, and a multi-physics fault assessment framework integrating mechanical response, corrosion evolution, and stress concentration analysis is established for quantitative fault grading and maintenance decision-making. Experiments on a real catenary dataset covering 10 component categories demonstrate that the proposed framework achieves a 0.953 image-level AUROC and a 0.957 pixel-level AUROC, outperforming existing methods while maintaining strong cross-component generalization and providing quantitative fault grading support for intelligent railway catenary maintenance.

## 1. Introduction

**Figure 1:**
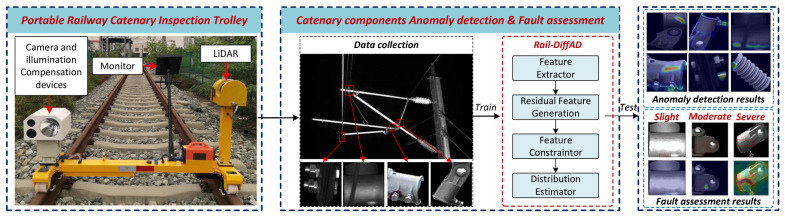
Industrial intelligent inspection system for railway catenary components: from portable data acquisition to diffusion model-driven anomaly detection and fault grading.

The catenary is a critical power supply component of electric railways (Figure 1), and its structural and electrical integrity directly determines the reliability of train power transmission [1,2,3,4]. Under harsh service conditions, long-term vibration, mechanical fatigue, and environmental erosion accelerate the performance degradation of catenary components, leading to typical defects such as insulator cracking, fastener loosening, and surface wear [5,6,7]. These defects not only degrade electrical transmission performance but also threaten the operational safety and maintenance reliability of high-speed railways.

Accurate identification and severity assessment of catenary anomalies are prerequisites for intelligent condition-based maintenance. However, practical inspection systems still face several challenges, including tiny localized defects, ambiguous boundaries between normal degradation and structural faults, scarce abnormal samples, and the lack of physically interpretable fault severity indicators. Existing vision-based methods mainly focus on anomaly localization while overlooking the evolution characteristics and operational risks associated with detected defects.

Currently, visual anomaly detection (VAD) methods are categorized into three main types: reconstruction-based, feature embedding-based, and distribution modeling-based approaches [8,9,10,11], which have been widely applied in industrial inspection [12,13,14,15].

**Reconstruction-based methods** (e.g., autoencoders, variational autoencoders, GANs) identify anomalies by reconstructing normal samples [16,17] and measuring input-reconstruction discrepancies [18]. These methods offer intuitive interpretability for homogeneous textures but fail to detect fine-grained catenary defects due to reconstruction loss-induced detail disappearance [19].

**Feature embedding-based approaches** (e.g., PaDiM, PatchCore) [20,21,22] leverage pre-trained deep features and similarity metrics for anomaly discrimination. They achieve high accuracy but suffer from performance degradation caused by intra-class feature variations and distribution shifts across catenary components.

**Distribution modeling-based techniques** (e.g., normalizing flow models) [23] estimate the probability density of normal features for rigorous anomaly localization. However, their strict invertibility constraints lead to high computational costs and limited scalability for multi-modal catenary scenarios.

Moreover, existing catenary inspection studies primarily focus on defect detection and classification, while insufficient attention has been paid to quantitative fault severity assessment and physically interpretable maintenance analysis. In practical railway systems, maintenance decisions depend not only on anomaly localization but also on the estimated impact of defects on structural stability, corrosion evolution, and operational safety. Therefore, establishing a unified framework that bridges visual anomaly features with physical fault assessment remains an important challenge for intelligent railway maintenance [24,25,26].

To address these challenges, this paper proposes an intelligent fault assessment framework for railway catenary systems based on a novel Railway Diffusion-based Anomaly Detection (Rail-DiffAD) model. The framework combines residual-guided anomaly representation, diffusion-based feature distribution modeling, and severity-aware fault assessment to achieve robust anomaly localization and physically interpretable condition evaluation. Furthermore, visual anomaly indicators are integrated with mechanical response and corrosion evolution mechanisms to support quantitative maintenance-oriented fault grading.

The main contributions of this work are as follows:

(1) A residual mapping-based feature learning mechanism is introduced to mitigate inter-category differences via normal sample alignment, highlight anomaly deviations, and improve cross-component generalization for railway catenary inspection.

(2) A multi-scale partial convolutional spatial-channel attention (MPSCA) module combined with a Log-barrier Bi-directional Constraint Loss (LBBCL) is proposed to enhance fine-grained anomaly localization and dynamically constrain feature distributions under complex industrial backgrounds.

(3) A severity-aware fault assessment strategy is established by integrating diffusion-guided anomaly evolution with visual anomaly indicators, enabling quantitative evaluation of contact force deviation, corrosion degradation, and structural failure risk for intelligent maintenance decision-making.

## 2. Related Work

### 2.1. Vision-Based Catenary Inspection and Anomaly Detection

Railway catenary components operate under long-term vibration, cyclic loading and environmental erosion, making them vulnerable to defects such as insulator cracking, clamp loosening, and contact wire wear. These defects usually exhibit tiny localized regions, ambiguous boundaries, and strong background interference, posing challenges for intelligent inspection.

Existing vision-based inspection approaches mainly include supervised object detection and unsupervised anomaly detection methods. Traditional detectors (e.g., Faster R-CNN, YOLO) [27,28] rely heavily on large-scale annotated defect datasets, which are difficult to obtain for random catenary faults [29]. In addition, repeated downsampling operations often weaken micro-texture details, reducing sensitivity to subtle defects.

Unsupervised and weakly supervised methods [30,31,32] alleviate annotation dependence by modeling normal feature distributions through reconstruction, feature embedding, or probabilistic estimation. However, their performance on catenary inspection is still limited by inter-component appearance variations, scarce abnormal samples, and complex outdoor environments.

Cross-component generalization is another major challenge [33,34]. Traditional single-class models [35,36] require separate training for different components, resulting in high deployment cost and poor adaptability. Meanwhile, generalized feature extractors and semantic models [37,38] often overlook fine-grained defects such as microcracks and early-stage corrosion. Therefore, achieving robust anomaly detection with strong cross-component adaptability remains an important issue for practical catenary inspection.

### 2.2. Fault Assessment and Maintenance-Oriented Analysis

Recent studies have increasingly focused on fault severity assessment and maintenance-oriented analysis for railway infrastructure. Traditional catenary maintenance mainly relies on manual inspection and periodic measurements of mechanical or electrical parameters, which are labor-intensive and difficult to support continuous monitoring [39,40,41,42].

Existing assessment methods mainly involve mechanical response analysis, corrosion evolution modeling, and structural reliability evaluation [43]. Mechanical studies analyze pantograph-catenary interaction through contact force and vibration variation, while corrosion and fracture studies investigate material degradation and crack propagation under long-term environmental exposure. However, these methods usually depend on manually measured physical parameters and are difficult to integrate with vision-based inspection results in practical railway scenarios.

Current vision-based approaches mainly provide qualitative anomaly localization, lacking quantitative severity evaluation and maintenance decision support. Therefore, integrating visual anomaly information with physical fault evolution mechanisms remains essential for intelligent catenary maintenance.

## 3. Method

**Figure 2:**
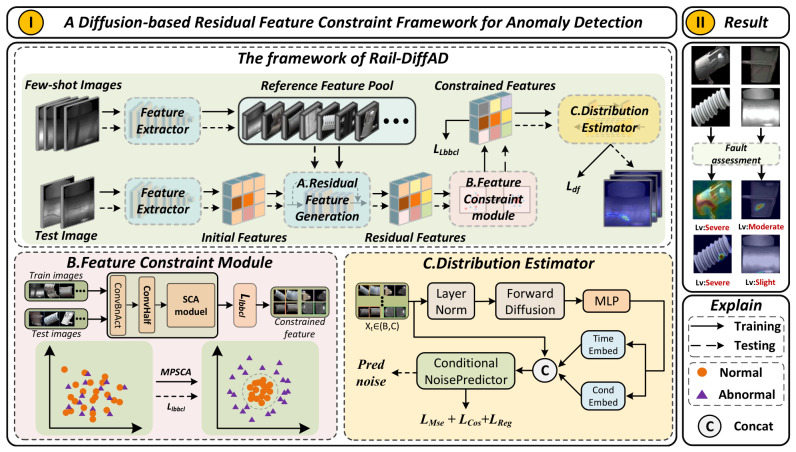
Rail -DiffAD Framework.

To address the challenges of catenary anomaly detection, this paper proposes Rail-DiffAD, a diffusion-driven fault identification framework integrating residual feature learning, feature constraint optimization, and diffusion-based distribution modeling. As shown in Figure 2, the framework combines a multi-scale partial convolutional spatial-channel attention (MPSCA) module with a log-barrier bidirectional constraint loss (LBBCL) to enhance anomaly-sensitive representations and suppress cross-category interference.

First, normal samples are used to construct a reference feature pool via a pre-trained feature extractor. Residual features are then generated through nearest-neighbor matching to suppress category-specific variations and highlight anomaly deviations.

Second, the residual features are refined within a radius-constrained latent space. LBBCL compactly constrains normal feature distributions while repelling abnormal features from the decision boundary, improving feature separability.

Finally, the refined features are fed into a conditional diffusion estimator, where progressive noise perturbation and reverse denoising are employed to model normal feature distributions and identify anomalous deviations.

### 3.1. Residual Feature Generation for Cross-Category Variation Mitigation

To mitigate category-specific patterns that weaken cross-component generalization, a feature learning approach based on residual mapping is introduced. Residual mapping suppresses category-specific patterns by comparing input features with normal references, thereby highlighting anomaly-related deviations. For a given input image $x\in R^{\left(H\times W\times 3\right)}$, we employ a pre-trained feature extraction network to extract features from multiple levels:

(1) $$F_{l}\left(x\right)\in R^{\left(C_{l}\times H_{l}\times W_{l}\right)},l=1,\ldots ,L$$

where $F_{l}\left(x\right)$ denotes the feature map extracted at layer *l*, and $C_{l},H_{l},W_{l}$ represent the number of channels, height, and width, respectively.

A reference feature pool composed of normal samples is constructed for each feature level.

For the feature vector $f_{l}{\left(x\right)}^{\left(h,w\right)}\in R^{C_{l}}$ at position (h,w) in the input image, we retrieve the most similar standard feature vector from the reference pool $M_{l}$:

(2) $$f_{l}^{ref}=argmin_{f\in M_{l}}\left|\right|f_{l}{\left(x\right)}^{\left(h,w\right)}-f{\left|\right|}_{2}$$

We then define its residual feature representation as

(3) $$R_{l}{\left(x\right)}^{\left(h,w\right)}=f_{l}{\left(x\right)}^{\left(h,w\right)}-f_{l}^{ref}$$

To further suppress redundant interference and enhance local anomaly responses, the MPSCA module is introduced for feature refinement.

### 3.2. Innovative Feature Refinement: MPSCA Module and LBBCL Loss

To further suppress complex background interference and benign texture fluctuations, a multi-scale partial convolutional spatial-channel attention (MPSCA) module is introduced.

#### 3.2.1. Multi-Scale Partial Convolutional Spatial-Channel Attention (MPSCA) Module

The HalfConv module applies local convolution to partial channels while preserving the remaining channels for global structure retention.

Since catenary anomalies are typically distributed in local regions, partial convolution enhances local anomaly sensitivity while preserving global structural information.

**Figure 3:**
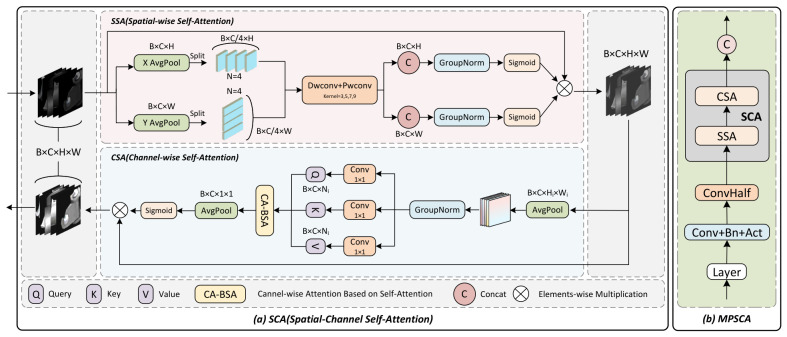
Structure Diagram of the MPSCA framework and spatial-channel attention mechanism.

The Spatial-Channel Attention (SCA) module (Figure 3a) synergizes Spatial-wise Self-Attention (SSA) and Channel-wise Self-Attention (CSA) to enhance critical spatial regions and channel-wise informative features simultaneously.

Spatial-wise self-attention (SSA) is introduced to enhance anomaly-sensitive regions under complex background interference. Specifically, directional pooling is first applied to the feature map [44]. Multi-scale convolution and directional pooling are jointly adopted to enhance anomaly-sensitive spatial dependencies.

Channel-wise self-attention (CSA) further enhances cross-component feature discrimination by adaptively modeling channel dependencies.

Together, SSA and CSA refine residual features into more discriminative representations for subsequent diffusion-based anomaly modeling [13,14,45].

#### 3.2.2. Log-Barrier Bi-Directional Constraint Loss

To further enhance the spatial separability of normal and abnormal residual features, we propose the Log-Barrier Bi-directional Constraint Loss (LBBCL). LBBCL constructs a soft hypersphere boundary in latent space, where normal features are compactly constrained while abnormal features are repelled outward to enhance feature separability.

Let the input feature vector be $f_{i}\in R^{d}$, and the sample mask $m_{i}$ is defined as follows: $m_{i}=0$ (normal sample), $m_{i}=1$ (abnormal sample). The feature radius $A_{i}$ is computed from the feature norm and used to characterize the distance between the sample feature and the normal feature center.

The logarithmic barrier constrains normal features within a compact radius interval. A logarithmic barrier function is introduced to penalize samples outside this interval:

(4) $$\begin{array}{c} L_{n}=E_{m_{i}=0}[-log\sigma \left(-\left(A_{i}-r_{max}\right)\right)\cdot e^{\left(A_{i}-r_{max}\right)}\\ -log\sigma \left(-\left(r_{min}-A_{i}\right)\right)\cdot e^{\left(r_{min}-A_{i}\right)}] \end{array}$$

where σ(·) is the sigmoid function.

Abnormal features are pushed away from the normal boundary while preserving structural consistency. The abnormal loss includes two terms: feature invariance and outward boundary constraint:

(5) $$\begin{array}{c} L_{a}=E_{m_{i}=1}[F_{i}-F_{i}^{target}{\left|\right|}_{2}^{2}+1-\cos\left(F_{i},F_{i}^{target}\right)\\ -log\sigma \left(-\left(r_{b}-A_{i}\right)\right)\cdot e^{\left(r_{b}-A_{i}\right)}] \end{array}$$

Here, $F_{i}^{target}$ is the target feature after abnormal sample space alignment; the first two terms form the anomaly invariance loss, while the last logarithmic barrier term pushes abnormal features away from the boundary ($r_{b}=r_{max}+\delta$, δ is a small constant, e.g., 0.05). The overall LBBCL is defined as

(6) $$L_{Lbbcl}=L_{n}+L_{a}$$

This enables separable feature distribution modeling in the latent space, providing high-quality constrained features for the subsequent diffusion-based feature distribution estimator.

It should be noted that the LBBCL loss leverages sample labels during the training stage to construct discriminative feature boundaries: normal samples ($m_{i}=0$) are compactly constrained within the hypersphere, while abnormal samples ($m_{i}=1$) are repelled outward. However, once trained, the model requires only a small set of normal reference samples to construct the feature pool for any new component category, without needing anomaly annotations on the target categories.

### 3.3. Diffusion Model-Based Feature Distribution Estimator for Anomaly Detection

To robustly model the complex feature distributions of catenary components, a diffusion-based feature distribution estimator is introduced.

As shown in Figure 2C, the proposed estimator progressively learns the latent distribution of normal features through iterative denoising, enabling stable anomaly discrimination under multi-modal catenary feature distributions [46].

#### 3.3.1. Forward Diffusion with Cosine Scheduling for Catenary Features

For the input feature vector $x\in R^{d}$, the forward diffusion process progressively perturbs features into Gaussian noise:

(7) $$q\left(x_{t}|x,t\right)=\sqrt{\alpha_{t}}x+\sqrt{1-\alpha_{t}}\epsilon ,\epsilon \in N\left(0,I\right)$$

where $t\in \left\{1,\ldots ,T\right\}$ denotes the diffusion time step, $\alpha_{t}=\prod_{s=1}^{t}\left(1-\beta_{s}\right)$ controls the noise attenuation rate, and $\beta_{s}$ is a noise intensity parameter that increases gradually.

A cosine scheduling strategy is adopted to stabilize noise evolution across different catenary feature distributions.

#### 3.3.2. Conditional Denoising Predictor with Transformer Backbone

**Figure 4:**
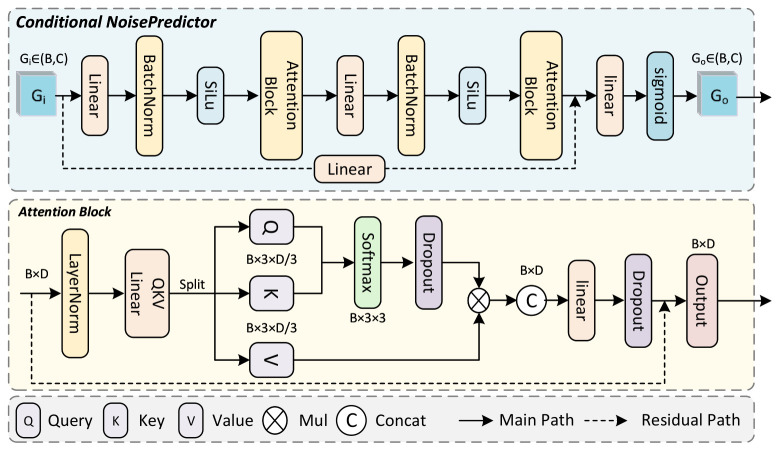
Framework diagram of the conditional noise predictor.

Based on the noisy feature distribution, a conditional denoising predictor (whose detailed structure is illustrated in Figure 4) is employed to estimate noise distributions under multi-category catenary priors.

A Transformer-based conditional interaction mechanism [47] is adopted to improve cross-component feature modeling, and a gating fusion strategy is introduced to enhance abnormal distribution deviations:

(8) $$\begin{array}{cc} \; & \tilde{x}=g⊙h\left(x,t,c\right)+\left(1-g\right)⊙r\left(x,t\right),\\ \; & g=\sigma (W[h(x,t,c),r(x,t)]) \end{array}$$

where h(·) represents the deep features extracted by the Transformer, r(·) is the residual branch, and *g* is the gating function.

The gating mechanism enhances abnormal distribution deviations while maintaining stable normal feature reconstruction.

#### 3.3.3. Multi-Component Loss for Fine-Grained Anomaly Sensitivity

To further improve the model’s ability to distinguish fine-grained anomalies, we designed a multi-component loss function to guide training, aligning with the noise prediction objective of the reverse denoising process:

(9) $$L_{\mathrm{df}}=\underset{\mathrm{MSE}}{\underset{︸}{\left|\right|\hat{\epsilon }-\epsilon {\left|\right|}_{2}^{2}}}+\lambda_{1}\underset{\mathrm{Cosine}}{\underset{︸}{\left(1-\cos\left(\hat{\epsilon }-\epsilon \right)\right)}}+\lambda_{2}\underset{\mathrm{Reg}}{\underset{︸}{\left|\right|\hat{\epsilon }{\left|\right|}_{2}^{2}}}$$

where $\hat{\epsilon }$ is the predicted noise, and ϵ is the true noise. MSE ensures the accuracy of noise prediction, Cosine Loss enhances directional consistency, and the regularization term is used to prevent overfitting. Meanwhile, we introduced category-adaptive weights, assigning higher weights to losses from abnormal samples to enhance sensitivity to fine-grained anomalies such as micro-cracks and stamp wear.

Through residual feature alignment, constrained feature refinement, and diffusion-based distribution estimation, Rail-DiffAD achieves robust anomaly characterization for multi-category catenary components and provides reliable anomaly representations for subsequent fault severity assessment.

### 3.4. Inference and Anomaly Scoring via Diffusion-Guided Noise Error

In the reasoning stage, after the input image is extracted with multi-level features by the encoder, it is matched with the normal features in the reference feature pool to generate residual features ${\tilde{e}}^{l}$; this residual feature further enhances the anomaly sensitivity through the feature constraint module and is then input into the diffusion model. During the forward diffusion and noise prediction process at time step *t*, normal features can be accurately restored by the diffusion model to the noise distribution, while abnormal features will cause significant prediction deviations. Based on this characteristic, the anomaly score is uniformly defined as the expected noise prediction error in multiple time steps:

(10) $$\begin{array}{c} S\left(x\right)=E_{l=1}^{L}E_{t∼U(1,T)}\left[\left|\right|{\hat{\epsilon }}_{\theta }\left(\sqrt{\bar{\alpha_{t}}}{\tilde{e}}^{l}+\sqrt{1-\bar{\alpha_{t}}}\epsilon ,t\right)-\epsilon {\left|\right|}_{2}^{2}\right] \end{array}$$

where ${\tilde{e}}^{l}$ is the residual feature of the *l*-th layer, ϵ∈N(0,I) is gaussian noise, and $\hat{\epsilon }$ is the noise prediction period of the diffusion model. Ultimately, the larger the score S(x), the higher the possibility that there are abnormal components in the input image.

### 3.5. Severity-Aware Diffusion Indicator for Fault Assessment

Although the anomaly score S(x) reflects the overall deviation between the predicted noise and the normal feature distribution, relying solely on averaged diffusion errors may overlook the temporal evolution characteristics of anomalies across diffusion steps. In practical railway catenary inspection, defects with different severity levels often exhibit distinct noise evolution patterns during the diffusion process.

To characterize the structural severity of anomalies, this paper further introduces a severity-aware diffusion indicator based on multi-time-step noise prediction trajectories:

(11) $$V\left(x\right)=\frac{\sum_{t=1}^{T}t\cdot \left|\right|{\hat{\epsilon }}_{t}-\epsilon_{t}{\left|\right|}_{2}^{2}}{\sum_{t=1}^{T}\left|\right|{\hat{\epsilon }}_{t}-\epsilon_{t}{\left|\right|}_{2}^{2}}\cdot S\left(x\right)$$

where ${\hat{\epsilon }}_{t}$ and $\epsilon_{t}$ denote the predicted and ground-truth noise at diffusion step *t*, respectively.

The proposed indicator measures the temporal centroid of diffusion reconstruction errors and reflects the evolution tendency of anomaly deviations throughout the diffusion process. Since larger diffusion steps correspond to higher-noise reconstruction stages, larger values of V(x) indicate that significant prediction errors persist even under severe diffusion perturbations, implying stronger structural inconsistencies and more severe defects. In contrast, smaller values are generally associated with shallow surface anomalies that introduce only limited structural disturbance. Thus, V(x) provides a severity-aware characterization that complements anomaly localization for downstream fault assessment.

### 3.6. Components Fault Assessment

Unlike conventional anomaly detection frameworks that only provide anomaly localization, Rail-DiffAD further utilizes diffusion-aware anomaly representations for downstream fault severity assessment and maintenance-oriented decision support.

The evolution of anomaly of catenary components is closely associated with the dynamic mechanical behavior of the pantograph-catenary system and the corrosion-induced aging of materials. Using the anomaly masks and anomaly scores output by Rail-DiffAD, a quantitative assessment model is established that integrates mechanical responses and corrosion damage. This model combines pantograph-catenary dynamics, corrosion kinetics, and fracture mechanics theories to theoretically link anomaly features with operational safety [48,49,50].

The coefficients in the following formulations are calibrated from industry standards and pantograph-catenary dynamics analysis, and their specific values and sources are presented in the experimental section later.

#### 3.6.1. Visual-Mechanical Coupled Fault Assessment

The stability of the contact force serves as a core indicator for evaluating the operational status of the catenary. Anomalies such as loosened positioning clamps and failed droppers can alter the suspension stiffness, leading to deviations in contact force. A surrogate contact-force deviation indicator is constructed by coupling visual anomaly features with mechanical priors:

(12) $$\begin{array}{c} \Delta F_{\mathrm{visual}}=k_{\alpha }\cdot V\left(x\right)+k_{\beta }\cdot \rho +k_{\gamma }\cdot \delta_{\mathrm{vis}} \end{array}$$

Here, $k_{\alpha }$, $k_{\beta }$, and $k_{\gamma }$ are weighting coefficients that quantify the contributions of structural defect severity, anomaly spatial extent, and geometric deformation to contact force deviation, respectively. These coefficients are calibrated from pantograph-catenary dynamic interaction analysis and catenary design standards, with their specific values given in Section 4.

V(x) denotes the severity-aware diffusion indicator, ρ is the spatial anomaly ratio from the predicted anomaly mask, and $\delta_{\mathrm{vis}}$ is the visually estimated structural deformation magnitude. Larger values of these three terms lead to larger $\Delta F_{\mathrm{visual}}$, reflecting more severe anomalies and greater contact force instability.

#### 3.6.2. Corrosion Damage Evolution

Metal components exposed to the outdoor environment for extended periods are prone to electrochemical corrosion, which leads to cross-sectional reduction and strength attenuation. This study integrates visual anomaly features into the corrosion evolution model to realize quantitative assessment:

(13) $$\begin{array}{c} D_{\mathrm{corr}}\left(t\right)=D_{0}\cdot e^{(k_{\mathrm{corr}}+k_{F}|\Delta F_{\mathrm{visual}}|)t}+\eta_{c}\cdot \rho \cdot V\left(x\right) \end{array}$$

In these expressions, $D_{\mathrm{corr}}\left(t\right)$ is the cumulative corrosion depth, $D_{0}$ is the initial corrosion depth, $k_{\mathrm{corr}}$ is the corrosion rate coefficient, and $\eta_{c}$ is an empirical corrosion acceleration coefficient that amplifies corrosion progression under severe visual anomalies.

The anomaly-aware term is introduced as an empirical degradation factor to characterize accelerated corrosion tendencies under severe structural anomalies.

The contact force deviation $\Delta F_{\mathrm{visual}}$ is introduced as a dynamic acceleration factor for corrosion evolution, reflecting the physical mechanism whereby unstable pantograph-catenary interaction intensifies vibration-induced material fatigue and corrosion propagation.

#### 3.6.3. Stress Concentration and Failure Risk Analysis

Under long-term dynamic loading, structural defects may induce local stress concentration and increase the risk of crack expansion and fracture. Based on a simplified engineering beam model, a correlation between visual crack features and mechanical stress is established:

(14) $$\begin{array}{c} \Delta \sigma_{\mathrm{vis}}=\frac{\Delta F_{\mathrm{visual}}\cdot L}{W\cdot (H-D_{\mathrm{corr}}\left(t\right))} \end{array}$$

Here, $\Delta \sigma_{\mathrm{vis}}$ denotes the equivalent stress amplitude estimated from visual anomaly indicators and corrosion-aware structural degradation. Larger corrosion depth $D_{\mathrm{corr}}\left(t\right)$ reduces the effective bearing area and further amplifies stress concentration around crack regions. Larger $\Delta F_{\mathrm{visual}}$ and deeper corrosion degradation lead to higher equivalent stress amplitudes, indicating increased structural failure risk.

This section establishes a quantitative fault assessment framework for catenary components through multi-physics visual-mechanical coupling models. By linking diffusion-based anomaly representations with mechanical response and corrosion evolution mechanisms, the framework extends anomaly detection toward physically interpretable service safety assessment and maintenance decision support. The proposed fault severity grading strategy further provides actionable quantitative guidance for intelligent catenary operation and maintenance.

## 4. Experimental Results and Analysis

**Figure 5:**
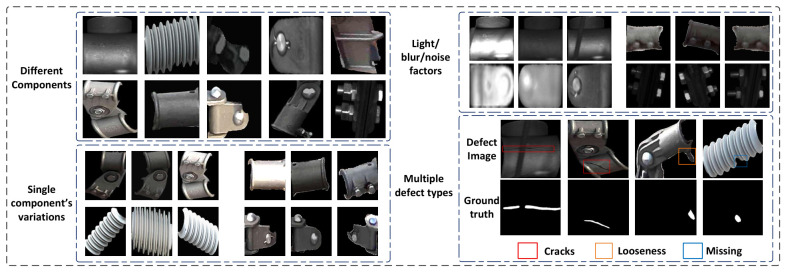
Examples of partial component images.

This section presents the experimental dataset, implementation details, evaluation metrics, and performance analysis. The effectiveness of Rail-DiffAD for railway catenary component anomaly detection is validated on a real-world catenary inspection dataset, as illustrated in Figure 5.

### 4.1. Experimental Setup

#### 4.1.1. Experimental Data and Parameter Settings

Original catenary images were acquired using a portable autonomous inspection trolley under operational railway conditions. Component regions were cropped from the raw images via a component localization model based on predicted bounding boxes, yielding individual component samples for subsequent annotation.

The dataset covers 10 typical catenary component categories with multiple defect types including cracks, loosening, and component missing. Representative categories such as casing base, insulator, locator base, sleeve double ear, and screw clamp were selected to cover diverse materials, scales, and structural types. Pixel-level ground-truth masks were generated using the Segment Anything Model (SAM), where each cropped component region was refined by SAM and padded with a black background to ensure annotation completeness. To enhance model robustness against complex interference, additional challenging samples containing stamp marks, uneven surface regions, and real fracture areas were incorporated. Detailed statistics are listed in Table 1.

The five categories selected for training are casing base, insulator, locator base, sleeve double ear, and screw clamp, which span distinct materials, scales, and structural types. The remaining five categories are reserved for testing. During training, only normal samples from the selected categories are used to learn the feature distribution of defect-free components, while both normal and abnormal samples from all 10 categories are used at test time to evaluate detection performance and cross-category generalization.

#### 4.1.2. Experimental Details

All images are resized to 224 × 224. WideResNet50 is adopted as the frozen feature extractor using features from layers [1,2,3]. The diffusion module employs a 3-layer Transformer Block with 4 attention heads and a hidden dimension of 256. The model is trained using the Adam optimizer for 100 epochs with a batch size of 96, a weight decay of $5\times 10^{-4}$, and an initial learning rate of $1\times 10^{-5}$, which decays after the 10th epoch.

The experimental software and hardware configuration are as follows: (1) CPU: Intel(R) Xeon(R) CPU E5-2697A v4, 2.60 GHz; (2) GPU: Nvidia Rtx4090 GPU; (3) Operating system: Ubuntu 22.04.3 LTS; (4) Code environment: Pytorch 2.0.0, Python 3.8.20; (5) Cuda version: CUDA-11.8, cuDNN-8.7.0.

#### 4.1.3. Experimental Evaluation Index

The anomaly detection task is formulated as a binary classification problem, where abnormal and normal samples correspond to positive and negative classes, respectively. Model performance is measured by Precision, Recall, and F1-score, which are defined as follows:

(15) $$Precision=\frac{TP}{\left(TP+FP\right)},Recall=\frac{TP}{\left(TP+FN\right)}$$

where TP, FP, and FN represent true positives, false positives, and false negatives, respectively. Additionally, the area under the receiver operating characteristic curve (AUROC) is adopted to evaluate the overall discrimination capability of the model under different decision thresholds. The AUROC characterizes the trade-off between the true positive rate (TPR) and false positive rate (FPR), where higher values indicate stronger separability between normal and abnormal samples.

### 4.2. Analysis of Experimental Results

#### 4.2.1. Comparative Analysis of Detection Performance

To fully verify the effectiveness of Rail-DiffAD in the anomaly detection task of railway catenary components, Table 2 presents a dedicated dataset covering 10 typical catenary components, with AUROC, AP, and F1-Score as the core evaluation metrics.

Performance comparisons were conducted with classic/advanced anomaly detection methods such as SSIM-AE, GANomaly, DRAEM, PatchCore, UniAD, and AA-CLIP, and all comparison methods adopted the original recommended parameters and training processes to ensure fairness. The average test results of the catenary components are shown in Table 2.

Table 2 compares Rail-DiffAD with representative anomaly detection methods on the proposed catenary dataset. Reconstruction-based methods (e.g., SSIM-AE and GANomaly) show relatively weak performance under large inter-category appearance variations and fine-grained defects. Discriminative methods such as DRAEM achieve improved localization ability but still exhibit limited robustness to complex catenary textures.

Compared with advanced baselines including UniAD and AA-CLIP, Rail-DiffAD achieves competitive or superior performance across most evaluation metrics, reaching 0.953/0.957 in I-AUC/P-AUC and 0.956/0.394 in I-AP/P-AP. Specifically, Rail-DiffAD surpasses UniAD by 2.4% in I-AUC, 3.1% in P-AUC, and 7.4% in P-AP, demonstrating the effectiveness of residual feature alignment and diffusion-based distribution modeling for pixel-level anomaly localization. It should be noted that UniAD achieves a marginally higher F1-score (0.929 vs. 0.917), indicating that Rail-DiffAD’s strength lies primarily in localization precision rather than image-level classification alone.

#### 4.2.2. Per-Component Detection Analysis

Table 3 presents the per-category detection performance of Rail-DiffAD across all 10 catenary component categories. The I-AUC ranges from 0.865 on the screw clamp to 0.997 on the connector sleeve, and the P-AUC from 0.867 to 0.995, yielding category-averaged scores of 0.953 and 0.957, respectively. Most categories achieve both an I-AUC and P-AUC above 0.94, while the comparatively modest scores on the screw clamp and the double ear can be attributed to the intrinsic challenge of resolving fine-grained defects on small metallic surfaces. Notably, the per-component metrics remain stable across a wide spectrum of component types, covering materials from ceramic to galvanized steel and geometries from tubular insulators to threaded fasteners, which substantiates the cross-category generalization capability of the proposed Rail-DiffAD framework.

#### 4.2.3. Computational Efficiency Analysis

Table 4 compares the computational complexity of Rail-DiffAD with baseline methods. All measurements were conducted on the same hardware platform with an input resolution of 224 × 224 pixels. Rail-DiffAD incurs a relatively long inference time of 631.0 ms per image and a large FLOPs count of 363.6 G, both of which arise from the 200-step iterative denoising process inherent to diffusion-based generative modeling. These computational overheads constitute the primary limitation of the proposed framework.

Future work will explore acceleration strategies to mitigate this limitation, including reducing denoising steps via DDIM sampling and applying model distillation techniques [51,52,53].

### 4.3. Ablation Studies

Table 5 presents an incremental ablation analysis evaluating the contributions of Rail-DiffAD’s core modules: Residual Features (RFs), the multi-scale partial convolutional spatial-channel attention module (MPSCA), and the Diffusion Model (DF).

A module-stacking strategy is adopted to isolate both the individual and synergistic impacts of each component.

Starting from DF alone, introducing RF improves the I-AUC/P-AUC by 6.2%/8.4%, validating its effectiveness in suppressing inter-category interference. Incorporating MPSCA further enhances fine-grained anomaly localization and background suppression, leading to consistent gains across all metrics. Combining RF, MPSCA, and DF achieves the best overall performance, demonstrating the complementary advantages of residual feature refinement, and diffusion-based distribution modeling.

In summary, stepwise performance gains validate the critical role of each module and their combined effect, confirming the rationality of the proposed framework design.

#### 4.3.1. Ablation Studies of RF

**Figure 6:**
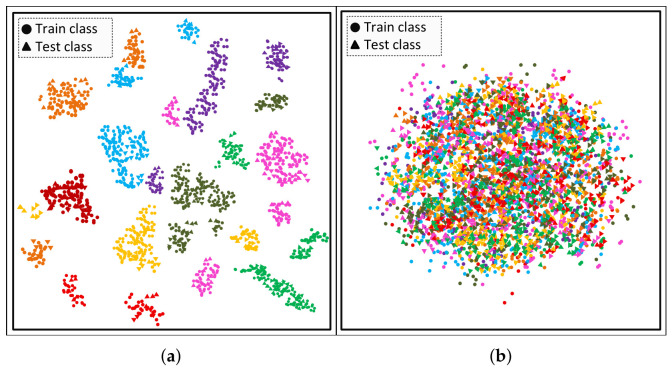
Residual Feature Extractor. (**a**) Initial Feature Distribution. (**b**) Residual Feature Distribution.

Figure 6 illustrates the effectiveness of the residual feature generation module within the Rail-DiffAD framework. The figure compares the feature distributions of various catenary component types before and after applying the residual feature generation process.

Figure 6 compares the feature distributions before and after introducing residual features. Before residual alignment, different component categories exhibit clear inter-class separation dominated by structural appearance differences. After residual feature extraction, inter-category distributions become more compact while anomaly-related deviations are preserved. These results demonstrate that the proposed RF module effectively suppresses category interference and enhances cross-category anomaly representation capability.

#### 4.3.2. Ablation Studies of MPSCA

To evaluate the effectiveness of each submodule in MPSCA, progressive component-stacking experiments are conducted on ConvBnAct, Convhalf, and SCA under the same training settings. The corresponding quantitative results are presented in Table 6.

The results indicate that Convhalf improves local anomaly sensitivity while preserving structural consistency, while the SCA module further enhances discriminative capability under complex backgrounds. The progressive improvements across all metrics validate the effectiveness of the proposed MPSCA design for fine-grained catenary anomaly detection.

#### 4.3.3. Ablation Studies of DF

**Figure 7:**
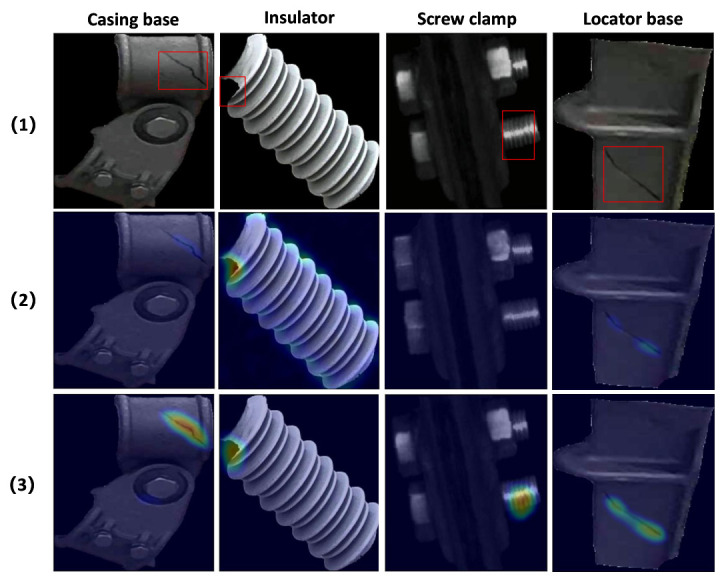
Performance Comparison of Different Models for Feature Distribution Estimator. The figure includes three regions: (1) Test image; (2) Results of RF + MPSCA + NF; (3) Results of RF + MPSCA + DF.

To evaluate the distribution modeling capability of the proposed DF module, comparative experiments with NF are conducted under the same RF and MPSCA setting. As shown in Table 7, replacing NF with DF improves I-AUC/P-AUC from 0.918/0.913 to 0.953/0.957 and I-AP/P-AP from 0.907/0.367 to 0.956/0.394. Figure 7 further confirms that DF produces clearer anomaly localization and more stable responses across different component types, particularly for small-scale structural defects. These results demonstrate the superior distribution modeling capability of diffusion-based feature estimation under complex catenary scenarios.

### 4.4. Abnormal Distribution and Score Analysis of Components

This study aims to quantify the anomaly severity of railway catenary components and verify the proposed model’s capability to model the correspondence between anomaly levels and feature spatial positions. Specifically, component anomaly levels are intuitively characterized via feature distributions in the hyperspherical space to facilitate accurate differentiation of catenary anomaly severity.

The experiment is conducted on a real railway catenary dataset, with abnormal samples annotated by severity levels in line with catenary industrial maintenance standards. Building on the model’s inherent feature extraction and spatial constraint mechanisms, the work focuses on analyzing spatial distribution patterns of abnormal samples across varying severity levels.

**Figure 8:**
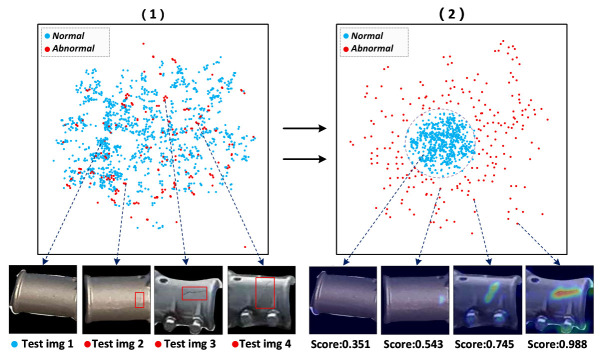
Abnormal Distribution and Score Analysis of Components: (1) overlapping normal/abnormal features & test samples; (2) clustered normal features, peripheral abnormal ones & anomaly scores.

As shown in Figure 8, experimental results reveal a strong correlation between hyperspherical feature distributions and catenary component anomaly severity. Features of normal samples closely cluster at the space center, forming well-demarcated feature clusters. In contrast, abnormal sample features distribute in peripheral regions, with their distance to the center varying gradually with anomaly severity.

Features of mild anomalies lie adjacent to normal feature cluster edges, while those of moderate anomalies locate in the space’s middle area. Severe anomaly features reside in the outermost region, with deviation distance from the normal center increasing progressively as anomaly severity intensifies.

**Figure 9:**
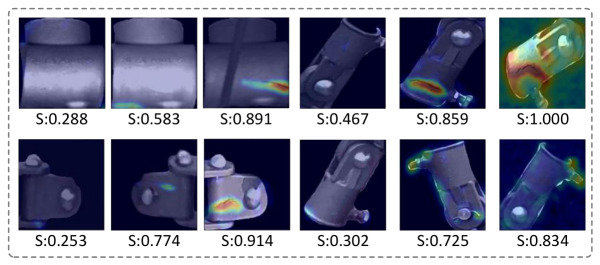
Anomaly score visualization of other catenary components.

This distribution pattern indicates that the model’s feature space naturally encodes severity information. Normal features cluster compactly at the center, while abnormal features reside in the periphery with a deviation distance increasing with defect severity. Figure 9 further visualizes the anomaly score maps across different component categories, showing that the model consistently assigns elevated scores to defect regions and suppresses background interference across diverse structural types and materials. These results provide a dual-dimensional verification of the model’s ability to distinguish anomaly severity levels through both feature-space distribution and pixel-level scoring, supporting differentiated maintenance prioritization.

### 4.5. Qualitative Visualization Results

**Figure 10:**
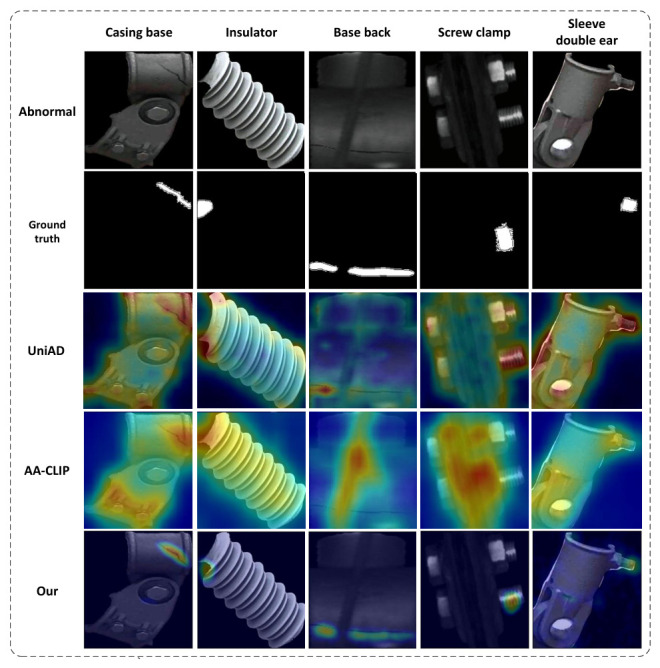
Qualitative visualization results.

Figure 10 shows the qualitative anomaly heatmaps of Rail-DiffAD on typical catenary defects. The model precisely locates defects including micro-cracks, loose bolts, and local wear, with heatmaps that align well with ground-truth annotations. It retains clear response boundaries even for subtle, low-contrast defects that are easily missed by reconstruction-based methods, and stably suppresses background noise under complex interference such as illumination fluctuation and texture diversity. Compared with traditional approaches, Rail-DiffAD yields more concentrated, noise-suppressed heatmaps with consistent localization across different component types.

### 4.6. Experimental Fault Grade Rating Table

The coefficients in the fault assessment models are calibrated from pantograph-catenary interaction standards [54,55], atmospheric corrosivity classifications [56,57], catenary component technical specifications [58], and corrosion experiment data [59]. A complete summary of all coefficient values, units, and calibration sources is provided in Table 8.

Based on these calibrated models, a multi-dimensional fault grade rating table is established in Table 9. The four grading indicators are all computed within the proposed framework: the anomaly score S(x) from Rail-DiffAD, the visually-derived contact force deviation $\Delta F_{\mathrm{visual}}$, the cumulative corrosion depth $D_{\mathrm{corr}}$, and the equivalent stress amplitude $\Delta \sigma_{\mathrm{vis}}$.

The $\Delta F_{\mathrm{visual}}$ thresholds are derived from the visual-mechanical coupling model (Equation (12)), where the coefficients $k_{\alpha }$, $k_{\beta }$, and $k_{\gamma }$ are calibrated from pantograph-catenary interaction standards EN 50367 [54] and TB 10621 [55]. The $D_{\mathrm{corr}}$ thresholds follow from the corrosion evolution model (Equation (13)), with the corrosion rate parameters referenced against ISO 9223/9224 [56,57] atmospheric corrosivity classifications and the initial galvanizing thickness specified in TB/T 2073 [58]. The $\Delta \sigma_{\mathrm{vis}}$ thresholds are determined by the equivalent stress model (Equation (14)), where the effective load-bearing thickness decreases with cumulative corrosion depth under contact force deviation. The S(x) boundaries are set to maintain internal consistency across all four grading dimensions, such that each fault grade corresponds to a coherent range of anomaly severity, mechanical deviation, corrosion progression, and structural stress.

As shown in Table 9, component health status is divided into four levels from normal to severe, each matched with targeted maintenance strategies. Severe anomalies require immediate shutdown and priority replacement, while slight and moderate faults trigger monitoring and planned maintenance, respectively.

**Figure 11:**
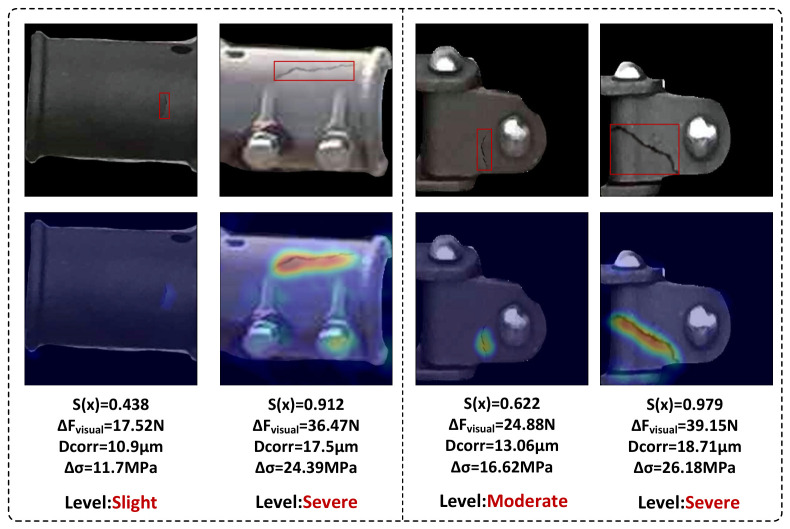
Visualization of catenary components fault grade assessment results.

Figure 11 visualizes the quantitative fault grade assessment results for typical catenary components. Each sample is evaluated through the computed indicators and classified into one of the four fault grades defined in Table 9, with higher-grade samples exhibiting larger $\Delta F_{\mathrm{visual}}$ and $D_{\mathrm{corr}}$ values. The results demonstrate that the proposed grading framework effectively distinguishes defect severity levels across different component types.

This section establishes a quantitative assessment framework integrating visual-mechanical coupling models with experimental fault rating criteria, forming a closed-loop from anomaly identification to maintenance decision-making.

## 5. Conclusions

Rail-DiffAD integrates residual feature learning with conditional diffusion-based distribution estimation, achieving reliable localization for fine-grained and ambiguous anomalies in complex catenary scenes. Experimental results on a real-world dataset demonstrate that the proposed method consistently outperforms existing state-of-the-art approaches in detection accuracy and pixel-level localization performance, while maintaining strong generalization across different component types.

Despite these improvements, several limitations remain. The model may still produce occasional false positives in densely textured regions, and its performance can be affected under extreme illumination or severe weather conditions. In addition, the overall computational cost is relatively high, which may limit real-time deployment on edge devices. Future work will focus on designing a lightweight variant of Rail-DiffAD [51,52,53,60,61], improving robustness in complex environments, and optimizing computational efficiency to better support onboard real-time catenary inspection systems.

## Figures and Tables

**Table 1:** The statistics of experimental data.

Categories	Training Set NS	Testing Set NS	Testing Set AS
Casing base	2388	301	125
Base back	764	1200	67
Connector sleeve	2328	224	206
Insulator	2824	246	158
Locator base	2452	248	147
Locator tube connector	3184	347	171
Screw clamp	2994	378	136
Sleeve double ear	3032	502	279
Sleeve loose	3628	388	149
Double ear	1084	1200	67

Note: NS denotes normal samples; AS represents abnormal samples.

**Table 2:** Comparative experiments of different anomaly detection methods.

Methods	I-AUC/P-AUC	I-AP/P-AP	F1-Score
SSIM-AE	0.722/0.725	0.787/0.234	0.787
GANomaly	0.774/0.764	0.825/0.257	0.815
DRAEM	0.847/0.853	0.862/0.313	0.871
PatchCore	0.893/0.885	0.903/0.305	0.897
UniAD	0.931/0.928	0.926/0.367	**0.929**
AA-CLIP	0.915/0.917	0.924/0.348	0.912
Ours	**0.953/0.957**	**0.956/0.394**	0.917

Note: Bold values denote the optimal results under each evaluation metric.

**Table 3:** Per-component detection performance.

Component Category	I-AUC/P-AUC	I-AP/P-AP	F1-Score
Casing base	0.983/0.992	0.990/0.550	0.945
Base back	0.985/0.988	0.988/0.520	0.950
Connector sleeve	0.997/0.995	0.996/0.480	0.990
Insulator	0.981/0.991	0.995/0.460	0.955
Locator base	0.950/0.958	0.955/0.399	0.914
Locator tube connector	0.986/0.986	0.998/0.409	0.961
Screw clamp	0.865/0.867	0.860/0.230	0.817
Sleeve double ear	0.941/0.940	0.930/0.240	0.880
Sleeve loose	0.972/0.970	0.950/0.280	0.900
Double ear	0.874/0.887	0.887/0.374	0.848
**Average**	**0.953/0.957**	**0.956/0.394**	**0.917**

Note: Bold values denote the average detection performance over all component categories.

**Table 4:** Comparison experiments of computational efficiency between different methods.

Methods	Params (M)	FLOPs (G)	Inference Time (ms)
SSIM-AE	85.3	234.1	52.4
Ganomaly	188.7	32.9	24.4
DRAEM	47.2	152.3	33.1
PatchCore	119.8	255.7	57.3
UniAD	24.7	3.9	23.0
AA-CLIP	397.2	1008.5	107.6
Rail-DiffAD (Ours)	95.2	363.6	631.0

**Table 5:** Incremental ablations.

RF	MPSCA	DF	I-AUC/P-AUC	I-AP/P-AP	F1-Score
**–**	**–**	✓	0.816/0.808	0.812/0.347	0.815
✓	**–**	✓	0.867/0.876	0.894/0.359	0.880
**–**	✓	✓	0.904/0.906	0.912/0.362	0.903
✓	✓	✓	**0.953/0.957**	**0.956/0.394**	**0.917**

Note: DF denotes diffusion model; RF represents residual feature. ✓ means the module is adopted, and ”–” means the module is removed. Bold values indicate the best performance in ablation experiments.

**Table 6:** Ablation study on the MPSCA module.

SCA	Convhalf	ConvBnAct	I-AUC/P-AUC	I-AP/P-AP	F1-Score
**–**	**–**	✓	0.872/0.864	0.866/0.321	0.884
**–**	✓	✓	0.895/0.896	0.913/0.349	0.907
✓	✓	✓	**0.953/0.957**	**0.956/0.394**	**0.917**

Note: ✓ indicates the module is adopted, and ”–” means the module is removed. Bold values represent the best performance.

**Table 7:** Ablation study on the DF module.

NF	DF	RF&MPSCA	I-AUC/P-AUC	I-AP/P-AP	F1-Score
✓	–	✓	0.918/0.913	0.907/0.367	0.891
–	✓	✓	**0.953/0.957**	**0.956/0.394**	**0.917**

Note: NF represents Normalizing Flow model. ✓ means the module is adopted, and ”–” means the module is removed. Bold values represent the best performance.

**Table 8:** Calibration summary of fault assessment coefficients.

Symbol	Value	Unit	Calibration Source
$k_{\alpha }$	25	N	Pantograph-catenary dynamics [54]
$k_{\beta }$	120	N	Catenary stiffness degradation [54,55]
$k_{\gamma }$	180	N/mm	Equivalent stiffness with projection correction [55]
$k_{\mathrm{corr}}$	$1.2\;\;\times \;\;10^{-4}$	day^−1^	ISO 9223 C2 corrosivity [56,57]
$k_{F}$	$3.6\;\;\times \;\;10^{-6}$	N^−1^day^−1^	Vibration-coupled corrosion [59]
$\eta_{c}$	0.12	mm	Damaged galvanized surface [58]
$D_{0}$	5	μm	Factory galvanizing baseline [58]
*L*	120	mm	Catenary fitting specification [58]
*W*	30	mm	Standard fastener cross-section [58]
*H*	6	mm	Nominal component thickness [58]

**Table 9:** Catenary component fault grade rating standard.

Fault Grade	Grade Definition	Core Indicators	O&M Strategy
Level 1 (Normal)	No obvious anomaly, intact performance	S(x)<0.3; $\Delta F_{\mathrm{visual}}\leq 12$ N; $D_{\mathrm{corr}}\left(10\mathrm{yr}\right)<10$ μm; $\Delta \sigma_{\mathrm{vis}}<8$ MPa	Routine inspection
Level 2 (Slight)	Slight anomaly, operation unaffected	0.3≤S(x)<0.5; $12<\Delta F_{\mathrm{visual}}\leq 20$ N; $10\leq D_{\mathrm{corr}}\left(10\mathrm{yr}\right)<12\;\mu m$; $8\leq \Delta \sigma_{\mathrm{vis}}<14$ MPa	Shorten inspection cycle
Level 3 (Moderate)	Obvious anomaly, potential failure risk	0.5≤S(x)<0.8; $20<\Delta F_{\mathrm{visual}}\leq 32$ N; $12\leq D_{\mathrm{corr}}\left(10\mathrm{yr}\right)<16\;\mu m$; $14\leq \Delta \sigma_{\mathrm{vis}}<21$ MPa	Enhanced tracking, formulate maintenance plan
Level 4 (Severe)	Severe anomaly, extremely high failure risk	S(x)≥0.8; $\Delta F_{\mathrm{visual}}>32$ N; $D_{\mathrm{corr}}\left(10\mathrm{yr}\right)\geq 16\;\mu m$; $\Delta \sigma_{\mathrm{vis}}\geq 21$ MPa	Immediate shutdown, priority replacement

## Data Availability

The data presented in this study are not publicly available due to privacy restrictions related to railway infrastructure. The data are available on request from the corresponding author for academic research purposes.

## References

[1] Hu Z., Chen L., Song Y., Liu Z., Pombo J., Antunes P. (2025). A deep learning-based surrogate model for dynamic interaction assessment of high-speed overhead conductor rail system. Eng. Struct..

[2] Wang X., Song Y., Wang X., Duan F., Liu Z. (2026). Study on Phase-Change Melting Characteristics of Rigid Catenary Wires under Dynamic Separation Process. Transp. Saf. Environ..

[3] Yan H., Lin C., Guo N., Xu Z., Zang J., Qing A. (2025). A three-stage framework for multi-type pantograph anomaly detection under complex environments. Comput. Electr. Eng..

[4] Yang H., Liu Z., Liu W., Wang H., Zhang Y., Wang H. (2026). Graph-MDETR: A Graph-Guided Mamba-DETR Network for UAV Catenary Support Components Detection in Electrified Railways. IEEE Trans. Intell. Transp. Syst..

[5] Chen J., Yin H., Zhang K., Ren Y., Zeng H. (2025). Integration of neural networks in brain–computer interface applications: Research frontiers and trend analysis based on Python. Eng. Appl. Artif. Intell..

[6] Wu X., Zou B., Lu C., Wang L., Zhang Y., Wang H. (2025). Dynamic security computing framework with zero trust based on privacy domain prevention and control theory. IEEE J. Sel. Areas Commun..

[7] Wei W.F., Zhang H., Xia L.Y., Luo Y.F., Zhou S.G., Huang G.Z., Yang Z.F., Wu G.N. (2024). Fatigue life enhancement of catenary droppers for high-speed railways based on arrangement optimization. Eng. Fail. Anal..

[8] Ma X., Wu J., Xue S., Yang J., Zhou C., Sheng Q.Z. (2023). A comprehensive survey on graph anomaly detection with deep learning. IEEE Trans. Knowl. Data Eng..

[9] Kesharwani A., Shukla P. A review of anomaly detection using machine learning techniques. Proceedings of the 2024 1st International Conference on Advanced Computing and Emerging Technologies (ACET).

[10] Ma N., Yang H., Liu Z., Cui H., Wang H., Shi L. (2026). Support Structure Detection of Railway Catenary Systems on UAVs Using Spike-Based Brain-Inspired Neural Network. IEEE Trans. Instrum. Meas..

[11] Hong Z., Guan S., Zhao Z., Zhang J., Li Z., Ma C., Yang L. Intelligent detection and analysis of brain tumors based on deep learning for CT scanning images. Proceedings of the 2026 7th International Seminar on Artificial Intelligence, Networking and Information Technology (AINIT).

[12] Zhang J., Xiang M., Hu Y., Hao W., Lei L., Yi K. (2026). Multivariate feature learning and associative spatial information enhancement for snow object detection in autonomous driving. Eng. Appl. Artif. Intell..

[13] Zhang J., Song X., Li Y., Liang D., Zhang Z., Cai J. (2026). Adaptive dual cross-attention network for multispectral object detection in autonomous driving. Expert Syst. Appl..

[14] Jiao R., Zhang J., Li C., Hu L. (2026). Large-kernel spatially parallel feature fusion for monocular 3D perception in autonomous driving. Knowl. Based Syst..

[15] Tian X., Xianyu X., Li Z., Xu T., Jia Y. (2024). Infrared and visible image fusion based on multi-level detail enhancement and generative adversarial network. Intell. Robot..

[16] Akcay S., Atapour-Abarghouei A., Breckon T.P. (2018). GANomaly: Semi-Supervised Anomaly Detection via Adversarial Training. Computer Vision—ACCV 2018.

[17] Lyu Y., Han Z., Zhong J., Li C., Liu Z. A GAN-based anomaly detection method for isoelectric line in high-speed railway. Proceedings of the 2019 IEEE International Instrumentation and Measurement Technology Conference (I2MTC).

[18] V. B.G., Deepa G., Febeena Ezhil Jothi S., Mahalakshmi L. (2025). Image Steganography with Security Using Massive Threefold Attentional Residual GAN Optimized by Chaotic PSO Algorithm. Cybern. Syst..

[19] Contreras-Cruz M.A., Correa-Tome F.E., Lopez-Padilla R., Ramirez-Paredes J.P. (2023). Generative adversarial networks for anomaly detection in aerial images. Comput. Electr. Eng..

[20] Defard T., Setkov A., Loesch A., Audigier R. (2021). PaDiM: A Patch Distribution Modeling Framework for Anomaly Detection and Localization. ICPR International Workshops and Challenges.

[21] Roth K., Pemula L., Zepeda J., Schölkopf B., Brox T., Gehler P. Towards Total Recall in Industrial Anomaly Detection. Proceedings of the 2022 IEEE/CVF Conference on Computer Vision and Pattern Recognition (CVPR).

[22] Tien T.D., Nguyen A.T., Tran N.H., Huy T.D., Duong S.T.M., Nguyen C.D.T. Revisiting reverse distillation for anomaly detection. Proceedings of the 2023 IEEE/CVF Conference on Computer Vision and Pattern Recognition (CVPR).

[23] Wu Y., Tao D., Zhan Y., Zhang C. (2022). BiN-Flow: Bidirectional normalizing flow for robust image dehazing. IEEE Trans. Image Process..

[24] Wu X., Dong J., Bao W., Zou B., Wang L., Wang H. (2024). Augmented intelligence of things for emergency vehicle secure trajectory prediction and task offloading. IEEE Internet Things J..

[25] Hu J., Chen D., Lei B., Sun J. Dynamic interaction-aware and causality-disentangled framework for multimodal sentiment analysis. Proceedings of the 2026 7th International Seminar on Artificial Intelligence, Networking and Information Technology (AINIT).

[26] Chen J., Shao Z., Zhu H., Chen Y., Li Y., Zeng Z., Yang Y., Wu J., Hu B. (2023). Sustainable interior design: A new approach to intelligent design and automated manufacturing based on Grasshopper. Comput. Ind. Eng..

[27] Duan F., Wang H., Yang H., Wei C., Zhang C., Song Y. (2025). MRFM-IFCOS: An Anchor-Free Interactive Detector Based on Multireceptive Field Mamba for Detecting Catenary Support Components. IEEE Trans. Instrum. Meas..

[28] Guo Q., Liu L., Xu W., Gong Y., Zhang X., Jing W. (2020). An improved faster R-CNN for high-speed railway dropper detection. IEEE Access.

[29] Divya U.H., Kumar J.P. (2025). Enhanced Transfer Learning-Based CNN for Abnormal Human Activity Detection in Video Surveillance Using Spatial-Temporal Features. Cybern. Syst..

[30] You Z., Cui L., Shen Y., Yang K., Lu X., Zheng Y., Le X. (2022). A Unified Model for Multi-class Anomaly Detection. Advances in Neural Information Processing Systems.

[31] Li C.L., Sohn K., Yoon J., Pfister T. CutPaste: Self-supervised learning for anomaly detection and localization. Proceedings of the 2021 IEEE/CVF Conference on Computer Vision and Pattern Recognition (CVPR).

[32] Yi J., Yoon S. Patch SVDD: Patch-level SVDD for anomaly detection and segmentation. Proceedings of the Asian Conference on Computer Vision (ACCV).

[33] Zhu S., Du B., Zhang L., Li X. (2022). Attention-based multiscale residual adaptation network for cross-scene classification. IEEE Trans. Geosci. Remote Sens..

[34] Zhang Y., Zhong J., Liu Z., Han Z. (2023). ECF-STPM: A robust crack detection method for railway catenary components. IEEE Trans. Instrum. Meas..

[35] Zavrtanik V., Kristan M., Skočaj D. DRÆM—A discriminatively trained reconstruction embedding for surface anomaly detection. Proceedings of the 2021 IEEE/CVF International Conference on Computer Vision (ICCV).

[36] Park E., Kim T., Kim M., Lee H., Lee G.J. SK-RD4AD: Skip-connected reverse distillation for robust one-class anomaly detection. Proceedings of the 2025 IEEE/CVF Conference on Computer Vision and Pattern Recognition Workshops (CVPRW).

[37] Chen X., Han Y., Zhang J. AA-CLIP: Enhancing Zero-shot Anomaly Detection via Anomaly-Aware CLIP. Proceedings of the Computer Vision and Pattern Recognition Conference.

[38] Yang H., Hu K., Wang H., Hong W., Wang X., Wang H., Song Y., Liu Z. (2026). BCLIP-ADer: A Bayesian Prompt Contrastive Language-Image Pretraining Method for Catenary Component Anomaly Detection in Electrified Railways. IEEE Trans. Transp. Electrif..

[39] Bobadilla H.A.F., Martin U. GAN-based Data Augmentation of Railway Track Irregularities for Fault Diagnosis. Proceedings of the 2024 International Joint Conference on Neural Networks (IJCNN).

[40] Yang G., Jiang Y., Wang S., Chen K. (2026). VinsFusion-Line: Binocular Vision Inertial Navigation Real-Time SLAM System Based on Line Features. Cybern. Syst..

[41] Shajeena J., Govindasamy B., Gnanasundaram M., Joel M.R. (2025). Mobile-Le Harmonic Fusion Network for Object Recognition and SiamMoT Based Multi-Object Tracking Using Video Surveillance. Cybern. Syst..

[42] Zhang M., Ma L., Wu Y., Shen K., Huang D., Leung H. (2026). Tackling the Kidnapped Robot Problem via Sparse Feasible Hypothesis Sampling and Reliable Batched Multistage Inference. IEEE Trans. Instrum. Meas..

[43] Fernández-Bobadilla H.A., Martin U. (2023). Modern Tendencies in Vehicle-Based Condition Monitoring of the Railway Track. IEEE Trans. Instrum. Meas..

[44] Qian C., Fong S., Yin H., Gao C., Qin H., Marques J.A.L. Design of a dual attention mechanism for small object detection. Proceedings of the 2025 7th International Symposium on Computational and Business Intelligence (ISCBI).

[45] Lin G., Yang S., Zheng W.-S., Li Z., Huang Z. (2025). A semantically guided and focused network for occluded person re-identification. IEEE Trans. Inf. Forensics Secur..

[46] Ho J., Jain A., Abbeel P. (2020). Denoising Diffusion Probabilistic Models. Advances in Neural Information Processing Systems.

[47] Qing L., Su B., Jung S., Lu L., Wang H., Xu X. (2024). Predicting human postures for manual material handling tasks using a conditional diffusion model. IEEE Trans. Hum.-Mach. Syst..

[48] Yao Y.M., Wang J., Xu Y., Wang B., Kou H.B., Zhou X.Y., Lu H.S. (2026). Image-based fatigue life prediction of catenary droppers in high-speed railways. Eng. Fail. Anal..

[49] Yan J., Zhou N., Cheng Y., Zhang F., Wang H., Wang M., Jin B., Li M., Lu Q., Zhang W. (2026). Application of Machine-Vision-Driven Physics-Informed Neural Networks in Pantograph–Catenary System State Detection. Mech. Syst. Signal Process..

[50] Chen D., Xu C., Yu J., Wang Q., Fang H., Yin S., Lookman T., Chen R. (2026). Interpretable machine learning framework for Nb–Si based alloy design with enhanced fracture toughness. Adv. Sci..

[51] Wang X., Yang X., Huang Z., Chen Y. (2026). CLIP-SD: CLIP-enhanced self-distillation for visual recognition. IEEE Trans. Multimed..

[52] Huang Z., Yang S., Zhou M., Li Z., Gong Z., Chen Y. (2022). Feature map distillation of thin nets for low-resolution object recognition. IEEE Trans. Image Process..

[53] Liu Z., Zhao W., Jia N., Liu X., Yang J. (2024). SANet: Scale-adaptive network for lightweight salient object detection. Intell. Robot..

[54] (2020). EN 50367: Railway Applications—Current Collection Systems—Technical Criteria for the Interaction Between Pantograph and Overhead Line.

[55] (2014). Code for Design of High-Speed Railway.

[56] (2012). Corrosion of Metals and Alloys—Corrosivity of Atmospheres—Classification, Determination and Estimation.

[57] (2012). Corrosion of Metals and Alloys—Corrosivity of Atmospheres—Guiding Values for the Corrosivity Categories.

[58] (2020). Technical Specification of Fittings for Overhead Contact SYSTEM in Electrification Railway.

[59] (2024). Technical Guidelines for Anti-Corrosion Operation and Maintenance Diagnosis Strategy of Power Grid Metal Equipment.

[60] Chen L. (2026). Beyond external constraints: The missing dimension of AI governance. SSRN Electron. J..

[61] Wu X., Wang H., Zhang Y., Zou B., Hong H. (2024). A tutorial-generating method for autonomous online learning. IEEE Trans. Learn. Technol..

